# Guava Fruit and *Acacia pennata* Vegetable Intake Association with Frailty of Older Adults in Northern Thailand

**DOI:** 10.3390/nu14061192

**Published:** 2022-03-11

**Authors:** Jetsada Ruangsuriya, Rawiwan Wongpoomchai, Somdet Srichairatanakool, Wachiranun Sirikul, Nida Buawangpong, Penprapa Siviroj

**Affiliations:** 1 Department of Biochemistry, Faculty of Medicine, Chiang Mai University, Chiang Mai 50200, Thailand; jetsada.ruang@cmu.ac.th (J.R.); rawiwan.wong@cmu.ac.th (R.W.); somdet.s@cmu.ac.th (S.S.); 2 Functional Food Research Center for Well-Being, Science and Technology Research Institute, Chiang Mai University, Chiang Mai 50200, Thailand; 3 Department of Community Medicine, Faculty of Medicine, Chiang Mai University, Chiang Mai 50200, Thailand; wachiranun.sir@cmu.ac.th; 4 Center of Data Analytics and Knowledge Synthesis for Health Care, Chiang Mai University, Chiang Mai 50200, Thailand; 5 Department of Family Medicine, Faculty of Medicine, Chiang Mai University, Chiang Mai 50200, Thailand; nida.buawangpong@cmu.ac.th

**Keywords:** fruits, guava fruit, vegetables, *Acacia pennata*, dietary consumption, frailty, older adults, Thailand

## Abstract

As Thailand moves toward an aging society, frailty has become a concern amongst northern Thai elderly. The causes of frailty are multifactorial and include genetic, environmental, and socio-economic factors; diet is of particular interest. A cross-sectional study was conducted from September to October 2017 to investigate what kind of diets normally consumed by 350 Thai elders were associated with frailty using a questionnaire and frailty determination by Fried’s phenotype followed by phytochemical analyses of the diets. The multivariable logistic regression analysis demonstrated a significant positive association between certain foods and lower frailty. Guava fruit and *Acacia pennata* vegetable consumption had lower odds of frailty, which were 0.52 times (95% CI 0.28–0.96, *p* = 0.037) and 0.42 times (95% CI 0.21–0.83, *p* = 0.012) when adjusted for the potential confounders. The phytochemical analyses of guava fruit showed a significantly higher amount of total flavonoids (*p* < 0.001), total phenolic compounds (*p* = 0.002), and antioxidant capacity, including DPPH (*p* < 0.001), ABTS (*p* < 0.001), and FRAP (*p* = 0.002) when compared to those of banana. *Acacia pennata* vegetable contained a significantly higher amount of total phenolic compounds (*p* = 0.012) when compared to those of lettuce. These findings may assist in health promotion programs of frailty prevention by encouraging an increase in consumption of either guava fruit or *Acacia pennata* vegetable among Thai elderly.

## 1. Introduction

Frailty is a geriatric syndrome, which has been defined as physiological function decline with increase in age, resulting from a cumulative decline that depletes homoeostatic reserves until minor stressor events trigger disproportionate changes in health status. It increases the risk of adverse health outcomes as reduced functional reserve, impairs multisystem function, and can increase the risk of disability or premature death. It also increases healthcare costs and other social welfare costs [1,2,3]. Frailty is widespread in many parts of the world and is expected to increase in all regions due to the emergence of aging societies. In 1996, of the US population who were 65 years old or older, the frailty prevalence was 7.8% with a 1.81-fold increase in the rate of having serious illness compared with non-frail older people [4]. The prevalence of frailty in Chinese and Cuban elders was found to be 3.9% and 51.4%, respectively [5]. Recently, a systematic analysis of frailty in 62 countries showed the overall frailty prevalence assessed by either physical phenotype (PP) or deficit accumulation model (frailty indicies, FI) was 12% by PP and 24% by FI and both methods suggested that frailty in females was more prevalent than males [6].

It is well known that frailty results from multiple factors and is dependent on time of factor exposure. Physiological reserves decrease with age; however, with frailty, this decrease is accelerated and homoeostatic systems start to fail, leading to the development of a chronic low-grade proinflammatory condition, which is a strong risk factor for frailty [7,8]. Previous studies have indicated that age, female gender, low education, poor socioeconomic position, low physical activity, comorbidities, functional status, and nutritional status are all risk factors for frailty in older people in Asian countries [9,10,11,12]. Moreover, social conditions also play a crucial part of frailty development [13]. From a medical perspective, the crucial feature of frailty is that it is a dynamic process that can be prevented and sometimes reversed to robustness [14,15]. However, a paucity of information on its modifiable contributors is the main obstacle to developing preventative and therapeutic approaches for frailty. One of the modifiable contributors in frailty is the nutritional status of the individual. It is understood that low energy fuel intake is associated with frailty [16]. In addition, an inadequate intake of nutrients in each group of the food pyramid potentially shift the functional decline from independence to dependence in older adults [17]. Gaillard et al. suggested that the resting energy expenditure (REE) is 20 kcal/kg/day in healthy elders but 28 kcal/kg/day in frail elders in order to maintain life [18].

Dietary proteins have been heavily studied in frail elders. Reduced protein consumption was strongly associated with the loss of muscle, which resulted in loss of body weight and weakness [19]. The expression of genes involved in muscle protein synthesis was suppressed when there was an inadequate intake of dietary protein [20]. A longitudinal study of dietary protein intake over 3 years showed that the risk of frailty significantly dropped when there was a high amount of dietary protein intake [21]. However, frailty incidence was still reported in all groups of elders who have an intake over the standard protein intake at 0.7 g/kg/day [22]. Dietary protein itself is insufficient to maintain the non-frailty status of older adults.

Many research groups recently focused on fruit and vegetable intake (FVI) as a source of vitamins and minerals associated with frailty. High FVI (300–800 g/day) was inversely associated with frailty, while low FVI (50–200 g/day) was directly associated with frailty [23]. The low risk of frailty was associated with a high dose of FVI in a dose-dependent manner, and it was suggested that three portions of fruit and two portions of vegetables had the strongest association with a low risk of frailty [24]. A cohort study suggested that older women should have seven servings of FVI to be associated with low risk of frailty, while a portion with less than three servings of FVI was strongly associated with a high frailty risk [25]. Moreover, a meta-analysis indicated FVI was recommended to prevent a high risk of frailty [26]. Interestingly, FVI was not associated with the robustness or pre-frailty, suggesting that FVI intake was unable to reverse the frailty phenotype to pre-frailty or to non-frailty, but pre-frailty and frailty could be prevented from becoming non-frailty with sufficient amounts of FVI [27]. In addition to FVI, more specific dietary types have been examined. Mediterranean diets are described as the most healthy and contain many fresh vegetables, fruit and nuts, cereals, white meat, and olive oil [28]. Adherence to a Mediterranean diet is associated with a low risk of frailty [29,30]. Likewise, a systematic review and meta-analysis showed that the greater degree of adherence to the Mediterranean diet, the lower the incidence of the risk of frailty [31].

To put the Mediterranean diet into practice for elders around the world is not practicable; different countries have their own diets to be explored. For northern Thai older adults, everyday dishes contain many types of local vegetables. We conducted a cross-sectional study of the community-dwelling elderly in the north of Thailand to explore which specific types of fruits and vegetables are regularly consumed and their independent association with frailty. Furthermore, the total phenolic compounds, total flavonoids, and antioxidant capacity of commonly consumed fruits and vegetables related to frailty reduction were investigated to provide a mechanistic understanding of their frailty-prevention effect. The results from this study could be applied to health promotion programs for elders in both the northern and other parts of Thailand.

## 2. Materials and Methods

### 2.1. Study Design and Participants

This cross-sectional study was conducted during September to October 2017. Participants were older adults aged over 60 years who lived in the Pa Sang district, Lamphun Province in northern Thailand. Sample size was calculated using EpiInfo^TM^ version 7.2 [32] based on the population survey or descriptive study. We used the population size (84,924 persons) from the number of older adults in Lamphun Province in 2017. The sample size was calculated based on the prevalence of frail and prefrail older adults in Thailand (49.0%) [12], a confidence level of 95%, an acceptable margin of error of 5%, and a design effect of 1.0; 382 older adults were enrolled. Finally, the total sample size that completed the questions was 350 participants (91.6%). The inclusion criteria were participants living in the sampled villages for at least 6 months who agreed to participate in this study. The participants who were diagnosed with following illnesses were excluded: totally blind or deaf, bedridden, disability of both hands, severe joint inflammation, chest pain, heart diseases, dizziness, stroke, Parkinson’s disease, depression and cognitive impairment assessed by Mini-Mental State Examination-Thai version, MMST10 of less than 10 [33,34]. Lists of older adults were obtained from primary care unit records. Systematic random sampling was used to select the participants. In case of unavailability of the participant at the time of data collection, the participant in the next name list was selected.

After statistical analysis of the association between regularly consumed FVI and frailty, we conducted the biochemical analysis of the fruits and vegetables most frequently consumed (banana and lettuce) and guava fruit and *Acacia pennata* vegetable, which were significantly associated with reduced frailty for analysis of total phenolic compounds, total flavonoids, and antioxidant capacity. This analysis was carried out from February 2020 to July 2021.

### 2.2. Data Collection and Measurement

Face-to-face interviews were carried out, and the frailty status was assessed at the primary care unit, Pa Sang subdistrict primary care clinic, by the 5 public health workers who graduated with at least a Bachelor’s degree and had work experience in community health surveillance. A questionnaire was used to collect data on socio-demographic variables, e.g., age, gender, education level, marital status, occupation in the past, incomes, self-reported medical diagnoses such as hypertension, diabetes mellitus, cardiovascular disease, stroke, arthritis, osteoporosis, and chronic obstructive pulmonary disease (COPD), and frailty screening questions. In addition, the participants reported the five types of fruits and vegetables that they consumed the most often on a weekly basis in the past month. All measurements were standardized by the principal investigator.

#### 2.2.1. Assessment of Frailty

Frailty was defined using Fried’s phenotype [35], which is comprised of five criteria: weight loss, exhaustion, slowness, weakness, and low physical activity. Then, frailty was determined by considering all the criteria. Participants who met 3–5 criteria were frail; those with 1–2 were pre-frail. Participants who did not meet any criteria were non-frail:

(a) Unintended weight loss was indicated when older adults lost at least 4.5 kg over the past 12 months by self-reporting.
(b) Exhaustion was determined whether they felt exhausted using the following two statements: (a) I felt that everything I did was an effort; (b) I could not get going. The question asked was “How often in the last week did you feel this way?” 0 = rarely or none of the time (<1 day), 1 = some or a little of the time (1–2 days), 2 = a moderate amount of the time (3–4 days), or 3 = most of the time. The degree of exhaustion was further rated, and the score from 2 to 4 suggested a positive finding.
(c) Slowness was determined by walking along a 15-foot path and considered in conjunction with height and sex. A positive finding was suggested when they met one of the following criteria. Men with height ≤173 cm showed a walk time ≥7 s or with height >173 cm showed a walk time ≥6 s. Women with height ≤159 cm showed a walk time ≥7 s or with height >159 cm showed a walk time ≥6 s.
(d) Weakness was determined by grip strength of the non-dominant hand in relation to body mass index (BMI) and sex. Grip strength was measured using a handheld dynamometer (Takei TKK5001^®^). We used the handgrip criteria recommended by the consensus report of the Asian working group for sarcopenia [36]. A positive finding was suggested when the handgrip was less than 26 kg for men or less than 16 kg for women.
(e) Low physical activity was determined by self-reported frequency of engagement in activities requiring low to moderate levels of energy but was indicated when they performed the physical activities three times or fewer a month [37].

#### 2.2.2. Determination of Total Phenolic Compounds, Total Flavonoids, and Antioxidant Capacity of Fruits and Vegetables

The fruits and vegetables most frequently consumed (banana and lettuce) and those significantly associated between frail and non-frail groups (guava fruit and *Acacia pennata* vegetable) were analyzed for their contents of total phenolic compounds, total flavonoids, and antioxidant capacity. The ripe banana, unripe guava fruit, green fresh lettuce, and *Acacia pennata* vegetable were purchased from the local market in the study area, Pa Sang district. Prior to determination, 5 kg of the vegetables and the fruits were subjected to ethanol extraction using 15 liters of 95% ethanol. Rotatory evaporation was used to remove the ethanol from the extract and then lyophilized to obtain the extract powder by the freeze-drier (Labconco^TM^, Kansas City, MO, USA). The lyophilized powders of vegetables and fruits were examined for total phenolic compounds, total flavonoids, and antioxidant capacity.

Total phenolic content (TPC) and total flavonoid content (TFC) were assessed by the colorimetric method established by Punvittayagul et al. [38]. Briefly, total phenolic compounds in the extract were chemically reacted with Folin–Ciocalteau reagent and 7% (*w/v*) Na_2_CO_3_ solution with absorbance measurement at 765 nm. Gallic acid was used as the standard for calculation of TPC in the vegetables and fruits expressed in mg gallic acid equivalent (GEA)/g extract. Total flavonoids in the extract were chemically reacted with 5% (*w/v*) NaNO_2_, 10% (*w*/*v*) AlCl_3_∙6H_2_O, and 1M NaOH solutions with absorbance measurement at 532 nm. Catechin was used as the standard for calculation of TFC in the vegetables and fruits expressed in mg/g extract.

Three methods to determine antioxidant capacity of the vegetable and the fruit extracts were 2,2-diphenyl-1-picrylhydrazyl (DPPH), 2,2′-azino-bis-ethylbenzthiazoline-6-sulfonic acid (ABTS), and ferric-reducing antioxidant power (FRAP) assays [39]. Briefly, DPPH and ABTS assays were performed by mixing the extracts with DPPH and ABTS solutions for 30 and 4 min, and the absorbances at 517 and 734 nm were recorded respectively. Trolox (6-hydroxy-2,5,7,8-tetramethylchroman-2-carboxylic acid) was used as the standard, and the percentage of inhibition was calculated by the following equation. The results were expressed as %inhibition.

100 × (Absorbance at time 0-Absorbance at time 30 or 4 min)/Absorbance at time 0(1)

Unlike DPPH and ABTS assays, FRAP assay was performed in 10 mM 2,4,6-tripyridyl-S-triazine, 20 mM FeCl_3_, and 300 mM sodium acetate buffer (pH = 3.6) and after the extract incubation, the absorbance at 593 nm was recorded. Trolox was used as the standard, and the results were expressed in μM of Trolox equivalent.

### 2.3. Statistical Analysis

All analyses were performed using SPSS 22.0 (IBM, New York, NY, USA. The Kolmogorov–Smirnov test was used to assess the normality of distribution. Continuous variables with normal distribution were expressed as means with standard deviation (SD), while continuous variables with non-normal distribution were presented as medians with interquartile range. Categorical variables were described by the frequency with percentages. An independent Student’s *t*-test was used to compare continuous variables with normal distribution. A Mann–Whitney U test was used to compare continuous variables with non-normal distribution. A Chi-square test and Fisher’s exact test was used to compare categorical variables. Multivariable logistic regression models, which calculated the adjusted odds ratio (OR), and 95% confidence intervals were used to identify an independent association between the physical frailty status and the dietary intakes as well as the participants’ characteristics, including age, sex, marital status, incomes, and number of underlying diseases. All statistical analyses were two-sided, and a *p*-value < 0.05 was considered statistically significant.

## 3. Results

### 3.1. Characteristics of Study Population

The baseline characteristics of participants are presented in Table 1. The participants comprised 350 older adults; 56% of participants were found to be frail, and 44.0% were classified as non-frail. The overall mean age of the non-frail group (±SD) was 67.47 years (±5.70), and the mean age (±SD) of the frail group was 70.76 years (±7.51). Most participants were female (74.3%) and had only a primary education (60.6%). A minority-36.6% of older adults had no underlying disease. The majority of older adults with frailty were female (59.2%, *p* = 0.013), aged 75 years and over (67.4%), single status (70.0%), and had more than two underlying diseases (64.8%), especially hypertension, diabetes mellitus, and osteoporosis.

### 3.2. Prevalence of Physical Frailty in Older Adults

Table 2 shows the frailty phenotypes as observed in five of Fried’s frailty criteria. All five frailty indicators were analyzed on the categorical variables (weight loss, exhaustion, slow walking speed, and low grip strength) and continuous variables (weights, walking times, and grip strength). The results found that all variables were significantly different when compared between older adults with frailty and non-frailty.

### 3.3. Prevalence of Dietary Intake in Older Adults

**Figure 1:**
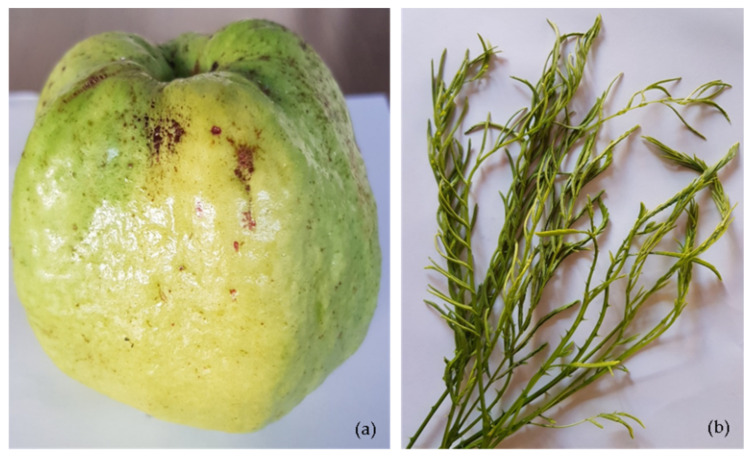
Frequent consumption of (**a**) guava fruit and (**b**) *Acacia pennata* vegetable by older adults living in northern Thailand was associated with reduced risk of physical frailty.

The participants reported the five fruits and vegetables that they consumed the most often on a weekly basis in the past month. Significant differences were only found between frail and non-frail groups in fruit and vegetable consumption (Table 3) while protein, carbohydrate, and lipid sources showed no association (Table S1). The most frequent fruit intake identified by older adults was banana (75.7%). The second and third most frequent were papaya (62.3%) and mango (49.4%), respectively. The least frequent fruit intake was guava fruit (17.1%) (Figure 1a); however, there was a significant association between the amount of guava fruit intake and frail and non-frail older adults (*p* = 0.005). The most commonly consumed vegetables were lettuce (87.4%), long bean (49.7%) and ivy gourd (44.6%) were the second and the third frequent, respectively. As with the fruits, *Acacia pennata* vegetable (Figure 1b) was the least frequently consumed vegetable and with a significant difference between frail and non-frail older adults (*p* = 0.013).

### 3.4. The Association between Fruit and Vegetable Intakes and Frailty in Older Adults

The multivariable logistic regression analysis demonstrates the independent association between food intake and physical frailty in older adults, as shown in Table 4. The results showed guava fruit and *Acacia pennata* vegetable consumption had lower odds of frailty, which were 0.52 times (95% CI 0.28 to 0.96, *p* = 0.037) and 0.42 times (95% CI 0.21 to 0.83, *p* = 0.012) when adjusted for the potential confounders. Older adults aged more than 75 years (aOR = 2.01, 95% CI 1.02 to 3.94, *p* = 0.042), married (aOR = 0.46, 95% CI 0.22 to 0.96, *p* = 0.038), and those with two underlying diseases (aOR = 2.07, 95% CI 1.15 to 3.73, *p* = 0.016) were associated with frailty.

### 3.5. Total Phenolic Compounds, Total Flavonoids, and Antioxidant Capacity of Certain Fruit and Vegetable Intakes

**Figure 2:**
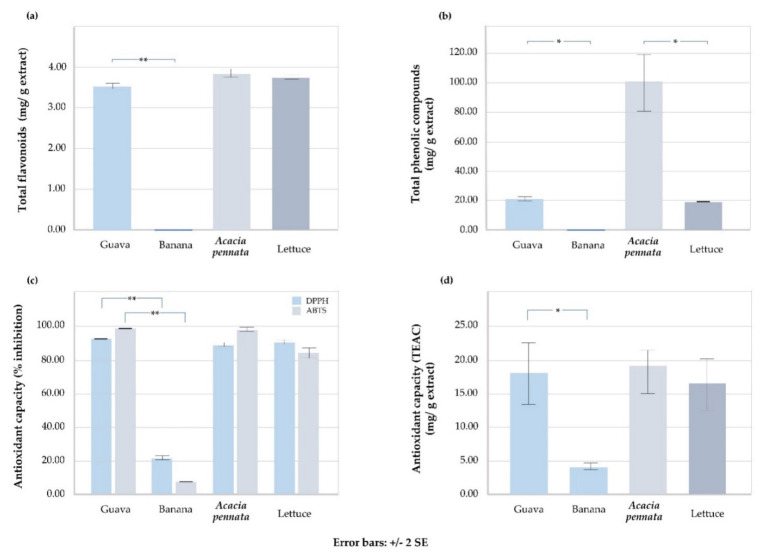
The flavonoid content, phenolic compounds, and antioxidant capacity levels of certain fruits and vegetables. Two fruits and two vegetables those are guava fruit, banana, *Acacia pennata*, and lettuce vegetables were analyzed for total flavonoids (**a**), total phenolic compounds (**b**), and total antioxidant capacity using 2,2-diphenyl-1-picrylhydrazyl (DPPH) assay, 2,2’-azino-bis (3-ethylbenzothiazoline-6-sulfonic acid) (ABTS) assay (**c**), and ferric-reducing antioxidant power (FRAP) assay (**d**). * and ** were significant at *p* < 0.05 and *p* < 0.001 analyzed by independent sample *t*-test, respectively.

Guava fruit had a significantly higher content of total flavonoids and phenolic compounds and antioxidant capacity than banana. Likewise, *Acacia pennata* contained a significant level of total phenolic compounds. Compared between fruits, banana, which was the most popular consumed fruit, contained neither phenolic compounds nor flavonoids while they were found to be 20.571 (±1.542) (*p* < 0.001) and 3.530 (±0.007) (*p* = 0.002) mg/g in guava fruit, respectively. Antioxidant capacity by DPPH, ABTS, and FRAP assays showed significantly higher levels in guava fruits. Likewise, compared between vegetables, *Acacia pennata* vegetable contained a significantly higher amount of total phenolic compounds at 100.603 (±15.623) (*p* = 0.012) mg/g, while lettuce contained significantly less (see Figure 2 and Table 5).

## 4. Discussion

Our investigation identified the association of regular consumption of guava fruit and *Acacia pennata* vegetable with a reduced frequency of physical frailty among northern Thai older adults. Of course, an increasing age is a key risk factor of the aging frailty, and there are still unknown parameters playing a role to promote or demote the frailty condition.

It is accepted that fruit and vegetable consumption has a strong association with a decrease in risk of frailty [40]. Mediterranean dishes as examples have been the food and methods of preparation accepted by Europeans. However, local Thai or other southeast Asian dwelling elders might not be able to put Mediterranean dishes into practice due to ingredient limitations. We have carried out this research and found what could be promoted in the diet of Thai elderly to prevent frailty: guava fruit and *Acacia pennata* vegetable.

Guava fruit, a tropical fruit with a scientific name of *Psidium guajava* L., has long been known as a source of ascorbic acid. In addition to vitamin C, dietary fibers, phenolic compounds, and antioxidant capacity were reported [41]. Our study found that regularly consuming guava fruit was associated with reduced frailty in elder participants. In addition, our investigation indicated that guava fruit contain significant amounts of total flavonoids, total phenolic compounds, and antioxidant capacity when compared with banana. The antioxidant activities of the guava fruit were consistent with those in the report of Thaipong et al. by FRAP assay; the antioxidant activity was directly correlated with the concentrations of ascorbic acid as well as total phenolic compounds in the fruit [42]. To promote the guava fruit consumption among elders might be difficult due to the hardness of the fruit texture and might be a reason why elders do not eat them if they have orthodontic problems. However, the consumption of the fully ripe guava fruits might not be as good as the unripe ones. It was found that the contents of total flavonoids, total phenolic compounds, and antioxidant capacity significantly declined from unripe to fully ripe guava, while the vitamin C content rose [43]. Although there have been plenty of documents reporting the medicinal effects of guava leaves, there have been few documents mentioning the guava fruits commonly consumed among Thai society. Only were the anti-hyperglycemic and anti-hyperlipidemia effects reported due to the dietary fiber and the active compounds in the extract playing a central role in guava fruits [44].

*Acacia pennata* vegetable, specifically *Acacia pennata* L. Willd subsp. *insuavis* (Lace) I.C. Nielsen, is a feathery bipinnate leaf plant of which the shoot tips are the most popular edible parts [45]. Our study found that regularly consuming *Acacia pennata* vegetable was associated with reduced frailty in elder participants. *Acacia pennata* vegetable contains significant amounts of total phenolic compounds when compared with lettuce. Our results were consistent with previous studies that *Acacia pennata* contained a high content of flavonoid and phenolic compounds [46,47]. *Acacia pennata* had various biological activities such as anti-nociception against pain [48], anti-inflammation through inhibition of cyclooxygenases-1 and -2 enzyme activities due to the flavonoid compositions [49], anti-cancer of pancreas and prostate [50], prevention of liver damage after acetaminophen-induced genotoxicity [51], and anti-Alzheimer disease by prevention of β-amyloid aggregation [52]. In addition, *Acacia pennata* crude extract at 245 mg/L could kill the larvae and pupae of *Aedes aegypti* mosquito, which is a potent dengue vector [53]. Due to the strongly specific metallic odor of *Acacia pennata*, it is unpleasant and not popular in the regular diet. Although many local dish recipes contain small amount of *Acacia pennata*, certain groups for whom *Acacia pennata* is not their favorite omit this part of the recipe. However, it is a good start to promote the consumption of *Acacia pennata* to prevent frailty with the addition of *Acacia pennata* into their dish.

We did not collect the frequency of consumption of the guava fruit and *Acacia pennata*, which could indicate exact consumption doses; instead, we can only estimate that respondents consumed both on a weekly basis during the past month, as indicated by the questionnaires. According to the 2019–2020 Thai National Health Survey, the median daily intake of FVI was 1 portion of fruit (equal to one medium-sized guava fruit) and two portions of vegetables for the northern Thai aged population [54]. Four medium-sized guava fruits and eight portions of *Acacia pennata* would be the minimum estimate of regularly consumed in a week in our study. There has been variation in reports on the recommended dose of FVI [23,24,25]; however, they were consistent in that the more the frequent elder’s intake, the less the prospect of frailty [29,30,31].

Our study demonstrated that regularly consuming guava fruit and *Acacia pennata* vegetable showed an independent association with reduced frailty regardless of the other candidate associated factors including age, gender, socio-economic status, and co-morbidities. Additionally, our investigation discovered that guava fruit and *Acacia pennata* vegetable contain significant amounts of total flavonoids and phenolic compounds. The well-established antioxidant properties of these phytochemicals may play a role in frailty prevention by maintaining antioxidant levels and resulting decreased inflammation [55,56,57].

Although the mechanism on how FVI can prevent physical frailty is still unclear, we suggest that a high degree of total antioxidant capacity in fruit and vegetables plays a crucial role in reduction in frailty prevalence in elders. It is well known that fruits and vegetables are the best sources of antioxidants. Diets containing a high total antioxidant capacity significantly reduced the prevalence of frailty in Japanese female elders [55] and the poor consumption of antioxidants, especially vitamin E, was significantly associated with the incidence of frailty in Australian male elders [56]. It is widely accepted that inflammation is one of the key factors in inducing frailty. The presence of significantly high levels of inflammatory cytokines, especially interleukin-6 (IL-6), tumor necrosis factor-alpha (TNF-α), and C-reactive protein (CRP) along with oxidative stress markers, particularly reactive oxygen species (ROS) are common in physical frailty [57]. In addition, other oxidative stress biomarkers were also reported such as malonaldehyde, 8-hydroxy-20-deoxyguanosine, reactive oxygen metabolites, and oxidized glutathione/glutathione along with the significant reduction in antioxidant levels in the body such as vitamin E and C [58]. However, these elevated biomarkers were not predictive indicators for physical frailty due to non-specificity [59]. Regular FVI of guava fruit and *Acacia pennata* are a source of antioxidants, which protect the oxidative stress of cells, tissues, and organs of the body, potentially preventing inflammatory processes and lowering the risk of physical frailty in the elderly.

Our findings were consistent with others that the frailty risk was significantly higher with increasing age [11,12,60]. We found that elders aged 75 years or older had a higher risk of frailty (around 2.10-fold more than those whose aged 60–64 years). Likewise, the prevalence of frailty in Swedish elders categorized as young-old age (65–74 years), old-old age (75–84 years), and oldest-old age (>85 years and above) increased from 15% in the first group to 36% in the last group [61]. It was not clear whether sex was associated with frailty. A previous study showed that females seemed to have more risk of frailty than males, but there was no difference when adjusted by multi-parameter analyses [10]. When frailty is assessed with a clinical or self-report based on frailty phenotype, females are frailer than males at all ages. Despite being frailer, women are at lower mortality risk. These sex-specific effects are likely resulting from a combination of behavioral, social, and biological factors, and they then have different consequences for disease susceptibility, treatment, and outcomes in frailty [62].

Another social parameter that plays an important role in frailty is marital status. We found that living alone was associated with a higher risk of frailty than living as a married couple. Our result was consistent with Progetto Veneto Anziani; frailty was found to be 3.84 times greater in single males and 1.43 times higher in widowed men than in married men. The lack and loss of a partner, particularly in men, may increase the development of frailty compared to those with a partner because having a partner might improve their lifestyle, nutritional condition, and health behaviors [63,64]. Recently, the meta-analysis of marital status and frailty also indicated that there was no significant difference in findings between men and woman [65].

It is reasonable that the greater the number of diseases any individual has, the higher the chance of frailty. We found that any elder that had two or more diseases had an association with frailty. The three diseases associated with frailty were hypertension, diabetes mellitus, and osteoporosis. Hypertension was considered as a common condition in frailty after the meta-analysis [66]. Hypertension was associated with frailty, which was found to be more prevalent among frail older adults with hypertension (83%) and estimated to have a 1.77 times higher risk of frailty than in older adults without hypertension [67]. Apart from hypertension, diabetes mellitus was found to be at a high prevalence in frail older patients whose physiological performance was impaired [68]. It was reported that the odds ratio toward frailty was 2.18 in diabetes mellitus patients due to unhealthy behaviors, obesity, and poor blood glucose control [69]. Sarcopenia is a well-known cause of frailty, unlike osteoporosis, which does not cause but impedes immobilization, leading to frailty. Both osteoporosis and frailty share common risk factors such as aging, sarcopenia, lack of physical activities, and low body weight due to the loss of hip and spine bone mineral density [70]. Osteoporosis patients have significantly low circulating osteogenic progenitor cells, which reduce during aging. This parameter was associated with frailty [71]. It was found that the prevalence of frailty in osteoporosis was 11.8%, while osteoporosis and sarcopenia known as osteosarcopenia showed greater prevalence of 29.1% [72]. Elders who suffered from osteosarcopenia had odds ratio toward frailty of 4.16 and 4.67 in men and women, respectively [73].

Two main limitations of this research are that the data from the questionnaire were based on self-reports and a cross-sectional study. We did not collect the frequency of consumption of each diet type, which reflects consumption doses, since collecting data on consumption frequency may be inaccurate and prone to recall bias, particularly when the participants are elderly.

The future studies should be conducted in a dose–response or absolute determination of how much each individual consumed each diet type. Due to financial constraints, the total antioxidant capacity in the plasma of all subjects as well as total antioxidant capacity with various methods and other nutritional components in all fruits and vegetables in Table 3 could not be performed.

## 5. Conclusions

Regular consumption of guava fruit and *Acacia pennata* vegetables was identified as having a decreased risk of physical frailty in northern Thai elderly. The high levels of total flavonoids, total phenolic compounds, and antioxidant content in guava fruit and *Acacia pennata* vegetable are the distinguishing feature of the foodstuffs and could account for the beneficial effect they have on reduced physical frailty in older adults. Their dose–response effect should be further determined to confirm their clinical effectiveness in frailty prevention.

## Figures and Tables

**Table 1:** Demographic and health characteristics of participants.

Characteristics	Total, *n* (%) (*n* = 350)	Physical Frailty Status, *n* (%)		
		Non-Frail (*n* = 154, 44.0%)	Frail (*n* = 196, 56%)	*p*-Value
Sex ^a^				
Male	90 (26.7)	48 (53.3)	42 (46.7)	0.013 *
Female	260 (74.3)	106 (40.8)	154 (59.2)	
Age, years ^a^, Mean ± SD	69.31 ± 6.96	67.47 ± 5.70	70.76 ± 7.51	
60–64	106 (30.3)	57 (53.8)	49 (46.2)	<0.001 **
65–74	158 (45.1)	69 (43.7)	89 (56.3)	
≥75	86 (24.6)	28 (32.6)	58 (67.4)	
Education ^a^				
No school	46 (13.1)	16 (34.8)	30 (65.2)	0.077
Primary school	212 (60.6)	89 (42.0)	123 (58.0)	
Secondary school	92 (26.3)	49 (53.3)	43 (46.7)	
Marital status ^a^				
Single	50 (14.3)	15 (30.0)	35 (70.0)	0.028 *
Married	171 (48.9)	86 (50.3)	85 (49.7)	
Widow/divorced/separated	129 (36.9)	53 (41.1)	76 (58.9)	
Occupation in the past ^a^				
Farmers	181 (52.9)	81 (44.8)	100 (55.2)	0.338
Merchants	85 (24.9)	40 (47.1)	45 (52.9)	
Official workers	33 (9.6)	17 (51.5)	16 (48.5)	
Housekeeper/unemployed	43 (12.6)	14 (32.6)	29 (67.4)	
Incomes, USD per month ^a^				
≤30	118 (33.7)	50 (42.4)	68 (57.6)	0.103
31–90	137 (39.1)	55 (40.1)	82 (59.9)	
91–180	52 (14.9)	31 (59.6)	21 (40.4)	
>180	43 (12.3)	18 (41.9)	25 (58.1)	
No. of underlying diseases ^a^				
None	128 (36.6)	67 (52.3)	61 (47.7)	0.035 *
1	131 (37.4)	55 (42.0)	76 (58.0)	
≥2	91 (26.0)	32 (35.2)	59 (64.8)	
Underlying diseases				
Hypertension ^a^	177 (49.4)	65 (36.7)	112 (63.3)	0.006 *
Diabetes mellitus ^a^	44 (12.6)	13 (29.5)	31 (70.5)	0.039 *
Cardiovascular diseases ^a^	17 (4.9)	8 (47.1)	9 (52.9)	0.794
Stroke ^b^	5 (1.4)	2 (40.0)	3 (60.0)	0.612
Arthritis ^a^	72 (20.6)	29 (40.3)	43 (59.7)	0.475
Osteoporosis ^a^	17 (4.9)	3 (17.6)	14 (82.4)	0.025 *
COPD ^b^	8 (2.3)	4 (50.0)	4 (50.0)	0.500

COPD, chronic obstructive pulmonary disease; Non-frail was indicated if the participant met none of the frail phenotypic criteria; Frail was indicated if the subject met 1 or more of the criteria; Significant *p*-values were analyzed by ^a^ Chi-square test, ^b^ Fisher’s exact test; * *p* < 0.05, ** *p* < 0.001.

**Table 2:** Comparison of physical frailty indicators between non-frail and frail older adults.

Parameters	Total (*n* = 350)	Non-Frail (*n* = 154)	Frail (*n* = 196)	*p*-Value
^a^ Weight (kg), Mean ± SD	54.11 ± 10.10	55.74 ± 9.41	52.83 ± 10.46	0.007 *
^b^ Unintended weight loss, n (%)	32 (9.1)	29 (90.6)	3 (9.4)	<0.001 **
^b^ Self-reported exhaustion, n (%)	7 (2.0)	0 (0.0)	7 (100.0)	0.016 *
^b^ Low activity, n (%)	63 (18.0)	62 (98.4)	1 (1.6)	<0.001 **
^c^ Walking time (sec), Mean ± SD	6.27 ± 2.29	5.17 ± 0.83	7.14 ± 2.67	<0.001 **
^b^ Slow walking speed, n (%)	112 (32.0)	1 (0.9)	111 (99.1)	<0.001 **
^c^ Grip strength (kg), Mean ± SD	22.59 ± 6.51	25.59 ± 5.74	20.23 ± 6.11	<0.001 **
^b^ Low grip strength, n (%)	102 (29.1)	0 (0.0)	102 (100.0)	<0.001 **

Significant *p*-values were analyzed by ^a^ Independent sample *t*-test, ^b^ Fisher’s exact test, ^c^ Mann–Whitney test; * *p* < 0.05, ** *p* < 0.001.

**Table 3:** Comparison of fruits and vegetables consumption in non-frail and frail older adults.

Types of Fruits and Vegetables ^≠^	Total (*n* = 350), *n* (%)	Non-Frail (*n* = 154) *n* (%)	Frail (*n* = 196) *n* (%)	*p*-Value
Fruits				
Banana	265 (75.7)	120 (45.3)	145 (54.7)	0.393
Papaya	218 (62.3)	92 (42.2)	126 (57.8)	0.384
Mango	173 (49.4)	76 (43.9)	97 (56.1)	0.979
Orange	153 (43.7)	67 (43.8)	86 (56.2)	0.945
Watermelon	128 (36.6)	58 (45.3)	70 (54.7)	0.707
Pineapple	122 (34.9)	61 (50.0)	61 (50.0)	0.098
Rambutan	122 (34.9)	46 (37.7)	76 (62.3)	0.083
Mangosteen	113 (32.3)	47 (41.6)	66 (58.4)	0.531
Durian	75 (21.5)	36 (48.0)	39 (52.0)	0.446
Guava	60 (17.1)	36 (60.0)	24 (40.0)	0.005 *
Vegetables				
Lettuce	306 (87.4)	134 (43.8)	172 (56.2)	0.835
Long bean	174 (49.7)	81 (46.6)	93 (53.4)	0.339
Ivy gourd	156 (44.6)	71 (45.5)	85 (54.5)	0.609
Eggplant	127 (36.3)	52 (40.9)	75 (59.1)	0.385
Morning glory	120 (34.3)	53 (44.2)	67 (55.8)	0.964
Gurmar (Local vegetable)	106 (30.3)	48 (45.3)	58 (54.7)	0.750
Cauliflower	94 (26.9)	34 (36.2)	60 (63.8)	0.074
Cucumber	67 (19.1)	31 (46.3)	36 (53.7)	0.677
Cabbage	67 (19.1)	34 (50.7)	33 (49.3)	0.216
Malabar spinach	58 (16.6)	20 (34.5)	38 (65.5)	0.110
*Melientha suavis*	58 (16.6)	19 (32.8)	39 (67.2)	0.059
Collard greens	52 (14.9)	18 (34.6)	34 (65.4)	0.140
*Acacia pennata*	44 (12.6)	27 (61.4)	17 (38.6)	0.013 *

≠ Frequently consumed types of fruits and vegetables in the last month; * Significant *p*-values were analyzed by Chi-square test; * *p* < 0.05.

**Table 4:** Association between guava fruit and *Acacia pennata* vegetable intakes with physical frailty in older adults.

	Frailty Status (Non-Frail/Frail)					
	cOR	95% CI	*p*-Value	aOR	95% CI	*p*-Value
Sex						
Male	Ref.			Ref.		
Female	1.66	1.03, 2.69	0.039 *	1.59	0.92, 2.79	0.099
Age, years						
60–64	Ref.			Ref.		
65–74	1.50	0.91, 2.46	0.108	1.26	0.74, 2.14	0.401
≥75	2.41	1.33, 4.35	0.004 *	2.01	1.02, 3.94	0.042 *
Marital status						
Single	Ref.			Ref.		
Married	0.42	0.22, 0.83	0.13 *	0.46	0.22, 0.96	0.038 *
Widow/divorced/separated	0.61	0.31, 1.24	0.172	0.56	0.27, 1.19	0.131
Incomes (USD per month)						
≤30	Ref.			Ref.		
30–90	1.10	0.66, 1.81	0.719	1.16	0.67, 2.01	0.600
91–180	0.50	0.26, 0.97	0.040 *	0.62	0.30, 1.27	0.189
>180	1.02	0.50, 2.07	0.954	1.44	0.65, 3.16	0.369
No. of underlying diseases						
None	Ref.			Ref.		
1	1.52	0.93, 2.48	0.096	1.43	0.84, 2.43	0.185
≥2	2.03	1.17, 3.52	0.012 *	2.07	1.15, 3.73	0.016 *
Regularly consumed						
Guava fruit	0.46	0.26, 0.81	0.007 *	0.52	0.28, 0.96	0.037 *
*Acacia pennata* vegetable	0.45	0.23, 0.85	0.015 *	0.42	0.21, 0.83	0.012 *

cOR, Crude OR values were analyzed with binary logistic regression; aOR, Adjusted OR values were analyzed with multivariate logistic regression; Confounding factors are sex, age, marital status, incomes and underlying diseases; OR = odds ratio; CI = confidence interval; Ref. = Reference; * *p* < 0.05.

**Table 5:** Mean and standard deviation of flavonoid content, phenolic compounds, and antioxidant capacity levels of certain fruits and vegetables.

	Total Flavonoids (mg/g)	Total Phenolic Compounds (mg/g)	Antioxidant Capacity		
			% Radical Scavenging		TEAC (mg/g)
			DPPH	ABTS	FRAP
Guava	3.530 ± 0.007	20.517 ± 1.542	91.893 ± 0.278	97.745 ± 0.247	17.850 ± 3.802
Banana	0.000 ± 0.000	0.000 ± 0.000	21.700 ± 1.380	6.845 ± 0.205	3.607 ± 0.692
*p*-value	<0.001 **	0.002 *	<0.001 **	<0.001	0.002 *
*Acacia pennata*	3.783 ± 0.146	100.603 ± 15.623	89.023 ± 1.589	96.480 ± 1.329	18.537 ± 2.879
Lettuce	3.650 ± 0.052	18.683 ± 0.032	91.437 ± 1.712	85.490 ± 3.663	16.53 ± 3.309
*p*-value	0.211	0.012 *	0.148	0.116	0.477

DPPH = 2,2-diphenyl-1-picrylhydrazyl; ABTS = 2,2’-azino-bis (3-ethylbenzothiazoline-6-sulfonic acid); TEAC = Trolox equivalent antioxidant capacity; FRAP = ferric-reducing antioxidant power; Significant *p*-values were analyzed by Independent sample *t*-test; * *p* < 0.05, ** *p* < 0.001.

## Data Availability

The data presented in this study are available on request from the correspondent author.

## Supplementary Materials

The following supporting information can be downloaded at https://www.mdpi.com/article/10.3390/nu14061192/s1, Table S1: Comparison of protein, carbohydrate, and lipid consumption in non-frail and frail older adults. File S1: Frailty assessment and dietary intake questionnaire among northern Thai elders.
