# Supplementary material for: Guava Fruit and Acacia pennata Vegetable Intake Association with Frailty of Older Adults in Northern Thailand
Source: Nutrients. 2022 Mar 11;14(6):1192. doi: 10.3390/nu14061192 (PMC8954598; doi:10.3390/nu14061192)
Supplement: Supplementary file 1 [file nutrients-14-01192-s001.zip › File S1.pdf]

Supplementary File S1. Frailty assessment and dietary intake questionnaire among Northern Thai elders:

There are 3 parts within this questionnaire: Part 1: General information, Part 2: Details of dietary intake and Part 3: Frailty assessment.

Part 1: General information

1. How old are you? .... years
2. Are you?
   Male
   Female
3. What is your educational level?
   No school
   Primary school
   Secondary school
4. What is your marital status?
   Single
   Married
   Widow/ divorced/ separated
5. What was your occupation in the past?
   Farmers
   Merchants
   Official workers
   Housekeeper
   Unemployed
6. How much your income per month (in Thai)?........................ Baht
7. Do you have any underlying diseases? Please choose all corresponding to you
   No
   Yes…. Hypertension
   Diabetes Mellitus
   Cardiovascular diseases
   Stroke
   Arthritis
   Osteoporosis
   Chronic obstructive pulmonary disease (COPD)
   Others (please specify) ………………………………

Part 2: Detail of dietary intake

1. What are sources of carbohydrates do you consumed the most often a weekly basis in the past month? (Excluded dietary fibers)
   Rice
   Others (please specify) ………………………………
2. What are sources of proteins do you consumed the most often a weekly basis in the past month? (Please choose 1-3 of them)
   Pork
   Beef
   Chicken
   Fish
   Buffalo
   Others (please specify) ………………………………
3. What are sources of plants-based proteins do you consumed the most often a weekly basis in the past month? (Please choose 1-3 of them)
   Peanut
   Bean
   Mushroom
   Others (please specify) ………………………………

4. What are egg protein sources you consumed the most often a weekly basis in the past month?
No
Yes
5. What are milk protein sources you consumed the most often a weekly basis in the past month?
No
Yes
6. What are vegetable oil sources you consumed the most often a weekly basis in the past month?
No
Yes
7. What are animal oil sources you consumed the most often a weekly basis in the past month?
No
Yes
8. What are types of fruits do you consumed the most often a weekly basis in the past month? (Please choose 1-5 of them)
Banana
Papaya
Mango
Orange
Pineapple
Guava
Watermelon
Rambutan
Mangosteen
Durian
Others (please specify) ………………………………
9. What are types of vegetables do you consumed the most often a weekly basis in the past month? (Please choose 1-5 of them)
Lettuce
Ivy gourd
Gurmar
Malabar spinach
Collard green
Long bean
Morning glory
Cabbage
Cauliflower
Eggplant
Cucumber
Acacia pennata
Melientha suavis
Others (please specify) ………………………………

Part 3: Frailty assessment
1. Unintended weight loss
Did you have unintended weight loss more than 4.5 kg in the past year?
No
Yes
2. Self-reported exhaustion
2.1 How often in the last week did you feel this way, "I felt everything I did was an effort"?
Rarely or none of the time (<1 day)
Some or a little of the time (1–2 days)
A moderate amount of the time (3–4 days)
Most of the time
2.2 How often in the last week did you feel this way, "I could not get going"?
Rarely or none of the time (<1 day)
Some or a little of the time (1–2 days)
A moderate amount of the time (3–4 days)
Most of the time

3. Slowness

Time spent for walking along 15-foot………… seconds

4. Weakness

Determined using a handheld dynamometer with non-dominant hand (the maximal force gained from 3 trials)

First …………. kg

Second……… kg

Third………… kg

5. Low physical activities

Do you have low or moderate physical activities such as gardening, car washing, or walking in a park?

Less than once a week

Once a week

1-3 times per month

Less than 1-3 times per month or never

References

1. Fried, L.P.; Tangen, C.M.; Walston, J.; Newman, A.B.; Hirsch, C.; Gottdiener, J.; Seeman, T.; Tracy, R.; Kop, W.J.; Burke, G.; et al. Frailty in older adults: evidence for a phenotype. *J. Gerontol. A Biol. Sci. Med. Sci.* **2001**, *56*, M146–M156, doi:10.1093/gerona/56.3.m146.

2. Danilovich, M.K.; Diaz, L.; Corcos, D.M.; Ciolino, J.D. Relationship between SHARE-FI Frailty Scores and Physical Performance Measures in Older Adult Medicaid Recipients. *Geriatrics (Basel)* **2018**, *3*, 51, doi:10.3390/geriatrics3030051.
