# Supplementary material for: Guava Fruit and Acacia pennata Vegetable Intake Association with Frailty of Older Adults in Northern Thailand
Source: Nutrients. 2022 Mar 11;14(6):1192. doi: 10.3390/nu14061192 (PMC8954598; doi:10.3390/nu14061192)
Supplement: Supplementary file 1 [file nutrients-14-01192-s001.zip › Table S1.pdf]

Table S1. Comparison of protien, carbohydrate, and lipid consumption in non-frail and frail older adults.

**Source of Macronutrients** ≠	**Total** (*n* = 350), *n* (%)	**Non-Frail** (*n* = 154) *n* (%)	**Frail** (*n* = 196) *n* (%)	***p*-Value**
Protien sources				
Eggs	328 (96.7)	148 (45.1)	180 (54.9)	0.103 [a]
Pork	241 (68.9)	100 (41.5)	141 (58.5)	0.160 [a]
Milk	184 (52.6)	78 (42.4)	106 (57.6)	0.523 [a]
Fish	91 (26.1)	45 (49.5)	46 (20.5)	0.210 [a]
Chicken	11 (3.2)	7 (63.6)	4 (36.4)	0.179 [a]
Beef/ buffalo meat	2 (0.6)	1 (50.0)	1 (50.0)	0.685 [b]
Others: peanuts/ bean/ mushroom	345 (98.6)	154 (44.6)	191 (55.4)	0.054 [b]
Carbohydrate sources (excluded dietary fibers)				
Rice ¥	350 (100.0)	-	-	-
Others: potato/ taro/ corn	223 (63.7)	104 (46.6)	119 (53.4)	0.188 [a]
Others: bread/ cake/ cookie	125 (35.7)	49 (39.2)	76 (60.8)	0.178 [a]
Lipid sources				
Vegetable oil	315 (90.0)	141 (44.8)	174 (55.2)	0.389 [a]
Animal oil	23 (6.6)	10 (43.5)	13 (56.5)	0.958 [a]

≠ Consumed the most often on a weekly basis in the past month; ¥ Rice is a staple food that Thai population eat often everyday or every meal. Significant *p*-value were analyzed by [a] Chi-square test, [b] Fisher's exact test.
